# Identification of key genes associated with anthracnose resistance in *Camellia sinensis*

**DOI:** 10.1371/journal.pone.0326325

**Published:** 2025-06-24

**Authors:** Li-Yi Xu, Jing-Jing Su, Cheng-Kang Zhang, Min Hao, Zi-Wei Zhou, Xiao-Hui Chen, Shi-Zhong Zheng

**Affiliations:** College of Biological Science and Engineering, Ningde Normal University, Ningde, China; Zhejiang A and F University, CHINA

## Abstract

Anthracnose, a prevalent fungal disease in tea plantations, cause substantial economic losses in tea production. Identifying resistance-associated genes in tea plants is crucial for developing anthracnose-resistant cultivars. This study used eight tea samples with differential anthracnose resistance for phenotypic evaluation, weighted gene co-expression network analysis (WGCNA) of RNA-seq data, WGCNA- QTL co-localization to identify resistance gene, and qRT-PCR validation of candidate genes. *in vitro* pathogen inoculation assay revealed that the lesion diameters of the eight samples ranged from 1.45 mm to 4.5 mm (ANOVA *p* = 4.4$\times 10^{-10}$). Using the ‘*Longjing 43*’ reference genome, transcriptome assembly achieved 93.9% gene detection rate (31,509/33,557 genes). WGCNA categorized expressed genes into 30 modules with the purple module (containing 907 genes) showing positive trait correlation and the yellow-green module (containing 781 genes) exhibiting negative correlation. Integration of WGCNA and QTL mapping identified two high-confidence candidate genes within LG08 QTL intervals. Both genes exhibited significant upregulation (t-test *p* < 0.01) in tea plant leaves following *Colletotrichum* spore inoculation. These findings provide actionable genetic targets for marker-assisted breeding of anthracnose-resistant tea cultivars.

## 1. Introduction

Tea plant [*Camellia sinensis* (L.) O. Kuntze], with their woody and perennial characteristics, hold significant economic value as the tea leaves they produce are one of the world’s three most popular non-alcoholic beverages [1]. Tea drinking originated from China, and now, it has become a daily habit for billions of people all over the world [2]. By the end of 2022, according to statistics from the Food and Agriculture Organization of the United Nations (FAO, https://www.fao.org/faostat/zh/#data), there were 46 countries and regions producing tea worldwide, China dominates the largest production with tea plant cultivating (3.39 million hectares) under tea cultivation yielding 14.53 million metric tons in the world. As a plant whose leaves are used for tea [3], anthracnose is a destructive leaf disease in tea plants that can result in substantial economic damage [4]. Anthracnose pathogens target parts of the tea plant such as the leaves, causing minor damage in the form of water-soaked lesions on leaf margins or tips, and severe damage leading to extensive defoliation and plant mortality, which significantly impacts both the quality and yield of tea [5]. For instance, the fungus *Colletotrichum fructicola* is responsible for estimated tea yield losses between 30% and 50% and has caused widespread defoliation in Guangdong Province, China [6]. The pathogen causing anthracnose in tea plants is a type of ascomycete fungus, and under favorable warm and humid conditions, various species within the genus can infect tea plants [7], leading to anthracnose and severe plant damage [8], such as *C. fructicola*, *C. gloeosporioides* [9]. Furthermore, in the major tea-growing regions of China, anthracnose pathogens can also induce secondary tea diseases such as tea leaf blight, tea brown blight [10].

At present, the management of diverse anthracnose pathogens in tea plants predominantly depends on foliar-applied chemical fungicides [11,12]. However, recurrent diseases outbreaks often lead to overuse of these chemicals, which, due to their residual toxicity, cause pollution issues affecting the health of humans and animals. It may also accelerate the emergence of fungicide-resistant pathogens strains, thereby exacerbating environmental and food safety risks. Consequently, a direct approach to addressing the issue is to develop resistant cultivars [13] and combine them with eco-friendly biological control strategies to help tea plants combat pathogens, minimizing chemical pesticides usage, which synergistically benefits ecological conservation and food security. As a perennial plant [14], traditional breeding methods for tea plants are time-consuming [15]. This necessitates in-depth exploration of key genes governing anthracnose resistance in tea plants and application of molecular breeding technologies for accelerate cultivar development. In such research, correlating phenotypic datasets with transcriptomic profiles enable us to rapidly identification of candidate genes for the desired traits. Weighted Gene Co-expression Network Analysis (WGCNA) is a genetic method that utilized large-scale gene expression matrices to decipher the correlations between genes, with particular efficacy in studying the relationships between functional modules to phenotypic traits [16,17]. By clustering tens of thousands of genes in the transcriptome dataset into discrete modules and associating them with target traits or phenotypes, the complexity of functional gene selection is reduced. WGCNA has been empirically validated for identifying co-expression modules and phenotype-relevant genes in tea plants [18–21].

Currently, researchers are focusing on the study of pathogens causing anthracnose in tea plants, especially the mechanisms by which tea plants respond immunologically to different invading pathogens [22–24]. Although preliminary explorations have been conducted at the molecular level, the majority of studies are focused on using RNA-Seq data from tea plants to uncover the regulatory mechanisms and relationships between gene expression and immune responses [25,26]. The resistance response of tea plants to anthracnose also involves multiple phytohormones. For instance, the expression of genes related to endogenous salicylic acid (SA) biosynthesis and the accumulation of SA in leaves are implicated in the tea plant’s response to *Colletotrichum* infection [27,28]. For instance, Li et al. discovered through transcriptomic and metabolomic analyses that genes associated with callose deposition and various plant hormone signaling pathways may play a crucial role following infection by anthracnose pathogens in tea plants [29]. The *CsUGT74B5* gene can fine-tune free SA levels by mediating SAG (SA glucoside) biosynthesis, thereby regulating tea plant immunity against anthracnose [30]. Additionally, the induction of the auxin receptor gene *CsAFB2* further activates defense-related genes, including pathogenesis-related (PR) genes and secondary metabolite biosynthesis genes, enhancing tea plant tolerance to *C. gloeosporioides* [31].

However, few studies have employed WGCNA to the identify anthracnose resistance-related genes in tea plants. In this study, we performed WGCNA using RNA-Seq data from eight samples, through which two modules significantly associated with anthracnose resistance were analyzed and identified. Furthermore, key phenotype-related genes were discovered via co-localization of WGCNA and QTL. This approach provided novel strategy for the functional gene screening in tea plants, and the identified genes underscore their potential significance in the breeding of resistant tea plant varieties.

## 2. Materials and methods

### 2.1. Plant material

In the experimental field, a two-year observational study was conducted on anthracnose susceptibility using the tea cultivars ‘*Longjing43*’ (♀, maternal parent) and ‘*Baihaozao*’ (♂, paternal parent), along with their F₁ hybrid population. Subsequently, six F₁ progeny with differential anthracnose susceptibility (based on field ratings) and their parental controls (Table 1) were selected for in vitro pathogen inoculation assays. Moreover, we selected ‘*Fuding Dabaicha*’ as the candidate gene validation material because it is commonly used as a reference cultivar in tea plant breeding programs, exhibiting superior agronomic performance.

**Table 1:** Characteristics of the nine tea plant cultivars/lines used in the experiment.

No.	Name	Relationship	Description
1	1103	F_1_	progeny lines of ‘LJ43’ × ‘BHZ’
2	1116	F_1_	progeny lines of ‘LJ43’ × ‘BHZ’
3	1509	F_1_	progeny lines of ‘LJ43’ × ‘BHZ’
4	1208	F_1_	progeny lines of ‘LJ43’ × ‘BHZ’
5	1305	F_1_	progeny lines of ‘LJ43’ × ‘BHZ’
6	1817	F_1_	progeny lines of ‘LJ43’ × ‘BHZ’
7	BHZ^1^	Paternal	nationally accredited tea plant cultivar
8	LJ43^2^	Maternal	nationally accredited tea plant cultivar
9	FD^3^	–	nationally accredited tea plant cultivar

^1^ Abbreviation of ‘*Baihaozao*’.

^2^ Abbreviation of ‘*Longjing 43*’.

^3^ Abbreviation of ‘*Fuding Dabaicha*’.

### 2.2. *In vitro* pathogen inoculation assay

Fungal spores were collected from symptomatic leaves and cultured on PDA medium. Following isolation and purification, the genomic DNA of pathogen was extracted using the DP305 Plant Genomic DNA Kit (TIANGEN, Beijing, China) for fungal species identification. The pathogen was identified by amplifying the ITS region using universal fungal primers ITS1/ITS4 (ITS1: TCCGTAGGTGAACCTGCGG, ITS4: TCCTCCGCTTATTGATATGC) [32].

An anthracnose spore suspension was prepared at a concentration of 1 × 10^6^ spores/mL using the identified pathogen for *in vitro* leaf inoculation. For each sample, ten uniform-sized healthy leaves were selected, wounded with a sterile insect pin, and inoculated with 10 μL spore suspension per wound. Inoculated leaves were maintained in a controlled-environment chamber (26 ± 1°C, 10,000 lux light intensity, 12/12 h light/ dark cycle) for disease development. Lesion diameters were measured 7 days post-inoculation using digital calipers [33]. The experiment was conducted with ten biological replicates per sample. Statistical significance was determined by one-way ANOVA (P < 0.01) followed by Tukey’s HSD test.

### 2.3. Transcriptome data processing

The transcriptome dataset PRJNA312027 was retrieved from the NCBI SRA database (https://www.ncbi.nlm.nih.gov/bioproject/PRJNA312027). The dataset comprised sequencing data from six progeny individuals and their parental controls (Table 1). Raw read quality was assessed using FastQC, and low-quality sequences (Q30 < 90%) were filtered. Using the cultivar ‘*Longjing 43*’ reference genome (GWH accession: GWHACFB00000000, CNCB, https://ngdc.cncb.ac.cn/gwh/Assembly/1086/show) as the alignment reference, sequence reads were assembled and aligned using Hisat2 [34]. Differentially expressed genes were identified using Stringtie [35].

### 2.4. Construction of weighted gene co-expression networks and identifying modules associated with anthracnose resistance

Initially, genes with excessive missing values were filtered out. Using R package (WGCNA) [36], thousands of genes were clustered into distinct modules (mergeCutHeight = 0.35, power = soft threshold). To evaluate the co-expression relationships between modules and anthracnose resistance phenotypes, we calculated the eigengene adjacency matrices based on their correlation coefficients. Heatmap visualization was performed to assess module-trait correlations, enabling identification of key anthracnose resistance-associated modules for subsequent key gene selection.

### 2.5. Localization of candidate module genes on the genetic map

To refine target gene selection, we mapped the genes from candidate modules onto our previously published high-density SNP genetic map [37]. This map was constructed using 2b-RAD technology [38], generating 27-bp marker sequences. Given this short read length, we performed alignments using Bowtie with parameters optimized “bowtie -f -a -v 2” for short sequences. To maximize mappable markers while addressing challenges from repetitive sequences in the tea plant genome, we implemented a two-tiered filtering strategy: 1) Prioritize marker sequences with Perfect matches; 2) Prioritize marker sequences with fewer mismatches (fewer than 3 mismatches) among Imperfect Matches.

### 2.6. Integrating WGCNA and QTL mapping for anthracnose resistance gene identification

We performed QTL mapping using the anthracnose resistance phenotypic data from our mapping population and the high-density SNP genetic linkage map [37]. Analysis parameters included: permutation test = 1000 repetitions, LOD threshold >3 at α = 0.05 level. Candidate genes were identified through co-localization of WGCNA-derived modules with QTL intervals. Since the reference genome (*Camellia sinensis* cv. ‘*Longjing 43*’) lacked functional gene annotations, we conducted functional prediction using EggNOG databases [39] (http://eggnog6.embl.de). This integrated approach enabled systematic prioritization of anthracnose resistance-associated genes in tea plants.

### 2.7. Validation and quantitative real-time PCR of candidate genes

To validate candidate genes identified through WGCNA-QTL integration, we conducted in vitro pathogen inoculation assays using leaves of cultivar ‘*Fuding Dabaicha’*. The experimental design included: sterile water treatment for 7 days (control group), anthracnose spore inoculation for 7 days (treatment group).

Total RNA was extracted from both infected and control leaves using the Column Plant RNA Extraction Kit (Sangon Biotech, China), followed by gDNA removal with MightyScript plus Master Mix (Sangon Biotech, China) and cDNA synthesis. Gene-specific primers were designed via Primer–BLAST. (Table 2)

**Table 2:** Summary of primers used in qRT-PCR.

Gene	Upstream primer	Downstream primer
*CsGAPDH*	TTGGCATCGTTGAGGGTCT	CAGTGGGAACACGGAAAGC
*GWHGACFB003565*	TGGTGAGGATGAGGCAAAGT	AGACTGGGGAAAGTAGCCAA
*GWHGACFB006749*	ACTATGAATCAGATGGCGGCAA	TTGCACACAGCGGCAATCTTT

Quantitative real-time PCR (qPCR) was performed on a QuantStudio 3 system (Thermo Fisher Scientific) using 2X HyperMB SYBR Green Master Mix (Sangon Biotech) with the following cycling protocol: (1) initial denaturation: 95°C for 15 sec, (2) amplification: 40 cycles of 95°C for 15 sec and 60°C for 30 sec. The *CsGAPDH* (Table 2) served as the internal reference. Relative gene expression was calculated using the 2^-ΔΔCt^ method. The experiment included three biological replicates and three technical replicates per biological sample. Statistical significance between control and treatment groups was assessed using Student’s t-test (p < 0.05) implemented in R (v4.2.0) with the stats package. Results were visualized using ggplot2, where asterisks denote significance levels.

## 3. Results

### 3.1. Phenotypic assessment of anthrax infection

Based on field evaluations of anthracnose disease incidence, six progeny lines with displaying differential resistance and the parental plants (‘*Longjing 43*’♀, ‘*Baihaozao*’♂) were selected for pathogen inoculation assays.

**Fig 1:**
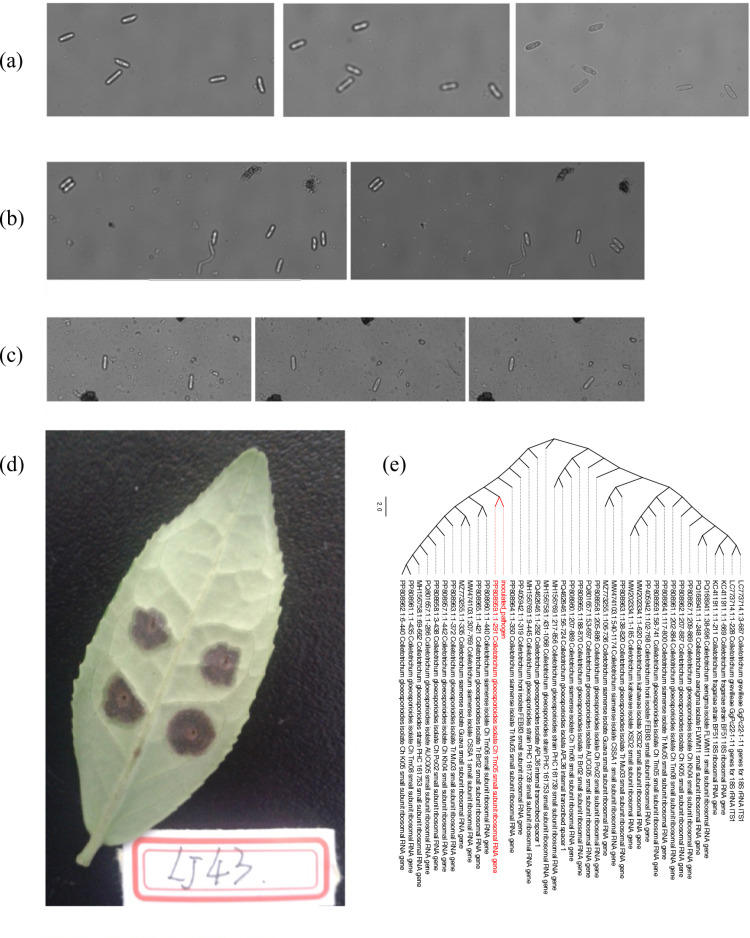
(a) The spore morphology of the pathogenic microorganism for inoculation. (b) & (c) The spore morphology on infected leaves 7 days post-inoculation. (d) The lesion morphology on infected leaves 7 days post-inoculation. (e) Phylogenetic tree constructed from the homology comparison of the inoculated pathogen’s ITS sequence.

Morphological analyses (Fig 1a–1c) demonstrated that the spore morphology of the pathogen used for inoculation is essentially identical to the spore morphology at 7 days post-inoculation. This confirms that the inoculated pathogen’s consistency with that derived from infected leaves. Additionally, the lesion characteristics on infected leaves at 7 days post-inoculation are consistent with those of tea anthracnose (Fig 1d). To validate the pathogen identity, we performed ITS sequencing of the inoculated strain. Upon sequencing and NCBI BLAST homology comparison (Fig 1e), we found the highest identity with *Colletotrichum gloeosporioides*, confirming it to be the anthracnose pathogen.

**Fig 2:**
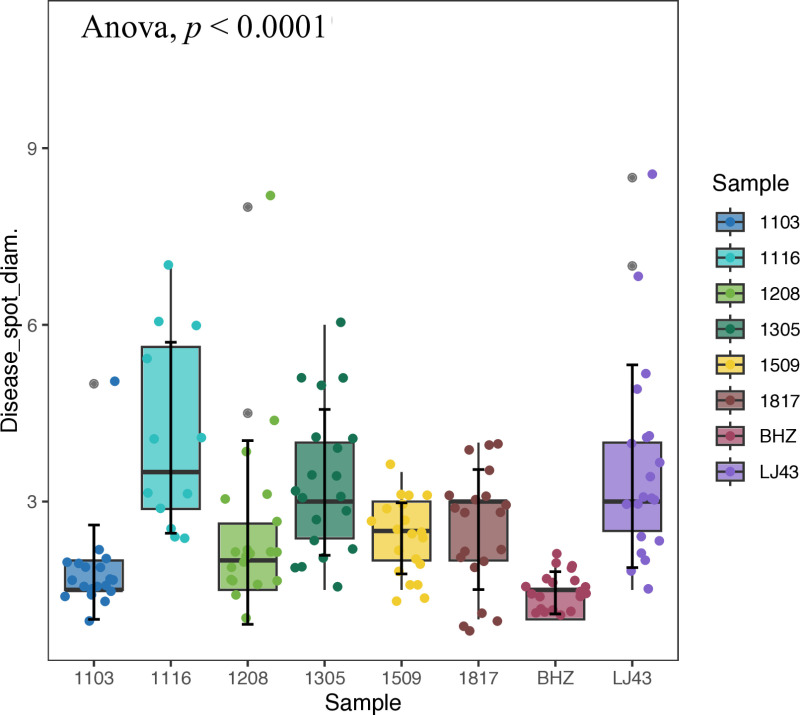
Boxplot of lesion diameter distribution in samples. A *p*-value of less than 0.01 indicates that there are significant differences among the samples.

At 7 days post-inoculation, lesions diameter of lesions was measured to evaluate sample. Statistical analysis of *in vitro* inoculation results showed that the mean diameter of the lesions formed by the pathogen infection was 1.45 mm to 4.50 mm (Fig 2). Parental plants ‘*Baihaozao*’ and ‘*Longjing 43*’ exhibited lesion diameters of 1.45 mm and 3.60 mm, respectively, aligning with the field observational study, indicating a significant difference between the parents. This difference is conducive to the formation of heterosis in the resistance phenotype of the offspring population. After the ANOVA for significant differences, it was found that there were significant differences in the size of lesion diameters among the samples (*p* = 4.4$\times 10^{-10}$).

### 3.2. Transcriptome data processing

Following quality control of raw transcriptome sequences, the samples exhibited original read counts ranged from 22,113,147–29,842,287 reads (Table 3). After quality filtering, more than 93% of high-quality sequences (Q = 30) were retained, yielding 20,730,753–28,246,256 reads. As all F_1_ samples are progeny lines of ‘Longjing 43’, its genome sequence served as the reference for aligning and annotating the transcriptome sequences of the samples (Table 4).

**Table 3:** The number of reads in the transcriptome of each sample before and after quality control.

Samples	Raw_reads	Clean_reads	Q30 (%)	GC (%)
1103	23566495	22073130	93.66	45
1116	22759423	21290470	93.55	44
1509	28681251	27125184	94.57	45
1208	24439144	22930798	93.83	44
1305	24475444	23168184	94.66	44
1817	22113147	20730753	93.75	44
BHZ^1^	28238157	26816962	94.97	44
LJ43^2^	29842287	28246256	94.65	44

^1^ Abbreviation of ‘*Baihaozao*’.

^2^ Abbreviation of ‘*Longjing 43*’.

**Table 4:** Sources of transcriptome and genomic data for the trial samples.

Samples	data_type	database	Accession No.
1103	transcriptome	NCBI_SRA	SRR3169844
1116	transcriptome	NCBI_SRA	SRR3180603
1509	transcriptome	NCBI_SRA	SRR3180604
1208	transcriptome	NCBI_SRA	SRR3180607
1305	transcriptome	NCBI_SRA	SRR3180610
1817	transcriptome	NCBI_SRA	SRR3180612
BHZ^1^	transcriptome	NCBI_SRA	SRR3180613
LJ43^2^	transcriptome	NCBI_SRA	SRR3180615
LJ43	genome	CNCB_GWH	GWHACFB00000000

^1^ Abbreviation of ‘*Baihaozao*’.

^2^ Abbreviation of ‘*Longjing 43*’.

**Fig 3:**
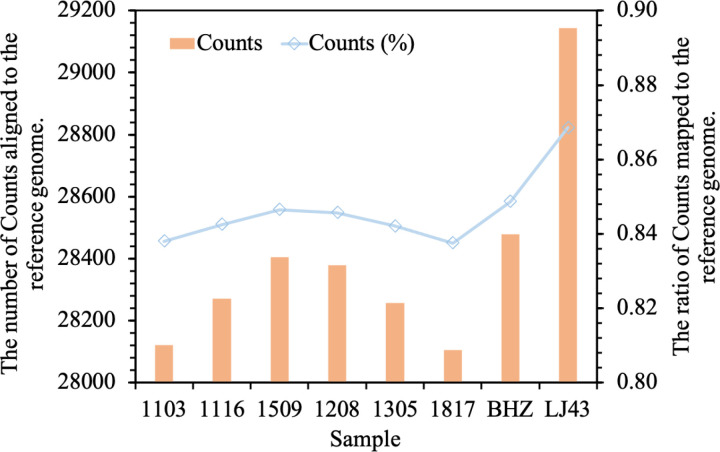
Gene expression abundance plot for samples. “counts” represents the number of reads from the samples that aligned to the ‘Longjing 43’ reference genome.

Using Hisat2 to constructed a reference genome index for ‘*Longjing 43*’ and aligned sample transcriptome reads to this index files, the gene expression abundance for each sample was estimated after assembly. The results (Fig 3) revealed that out of 33,556 gene sequences in the reference genome, 31,509 (93.9%) were detected with transcriptome reads, among which the number of reads detected in each sample ranged from 28,104 (83.75%) to 28,478 (84.87%).

### 3.3. Construction of weighted gene co-expression network

**Fig 4:**
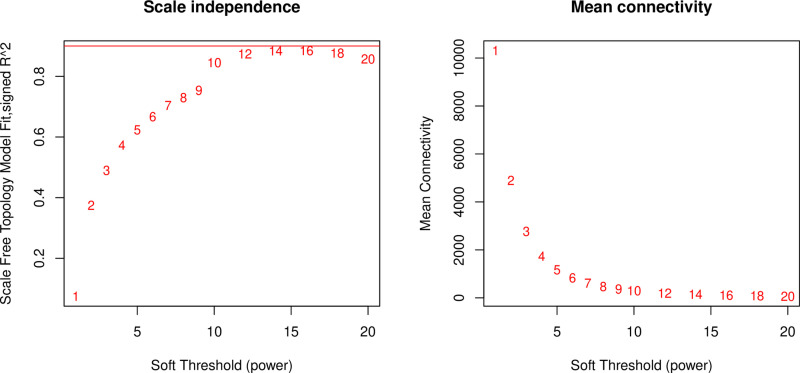
Network topologies for various soft-thresholding powers. The numbers in the plots cor-respond to the respective soft-thresholding powers. An approximate scale-free topology model fit of 0.9 can be achieved at a soft-thresholding power of 12.

**Fig 5:**
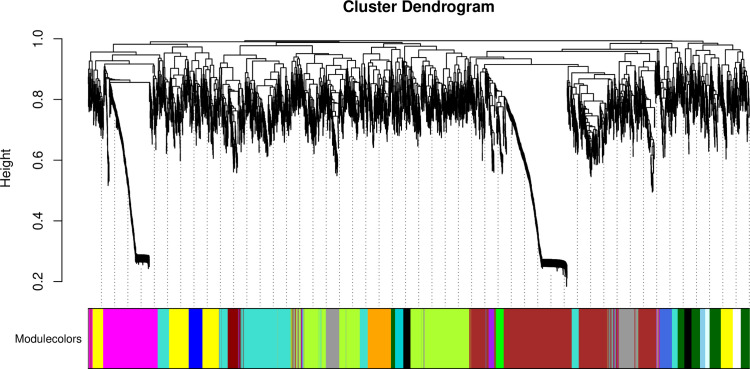
Gene modules identified by Weighted Gene Co-expression Network Analysis (WGCNA). The gene dendrogram was obtained by clustering based on the dissimilarity using consensus Topological Overlap, with the corresponding module colors indicated by the color row. Each colored row represents a color-coded module containing a group of highly connected genes. A total of 30 distinct modules were identified.

Using the gene expression abundances data from section 3.2, we constructed a gene expression matrix to calculate the scale-free topology fit index, which facilitated the selection of an appropriate soft-thresholding power. Analysis revealed that a soft-thresholding power of 12 achieved a scale-free topology fit index of 0.9 (Fig 4), which was subsequently adopted for building the WGCNA network. The network was constructed with following parameters: the minimum module size was 30 genes, the module detection sensitivity with deepSplit was 2, and the cut height for module merging was 0.35, which means that modules with a correlation higher than 0.65 would be merged. Ultimately, this process yielded a co-expression network comprising 30 distinct modules (Fig 5).

### 3.4. Module-trait correlation analysis

**Fig 6:**
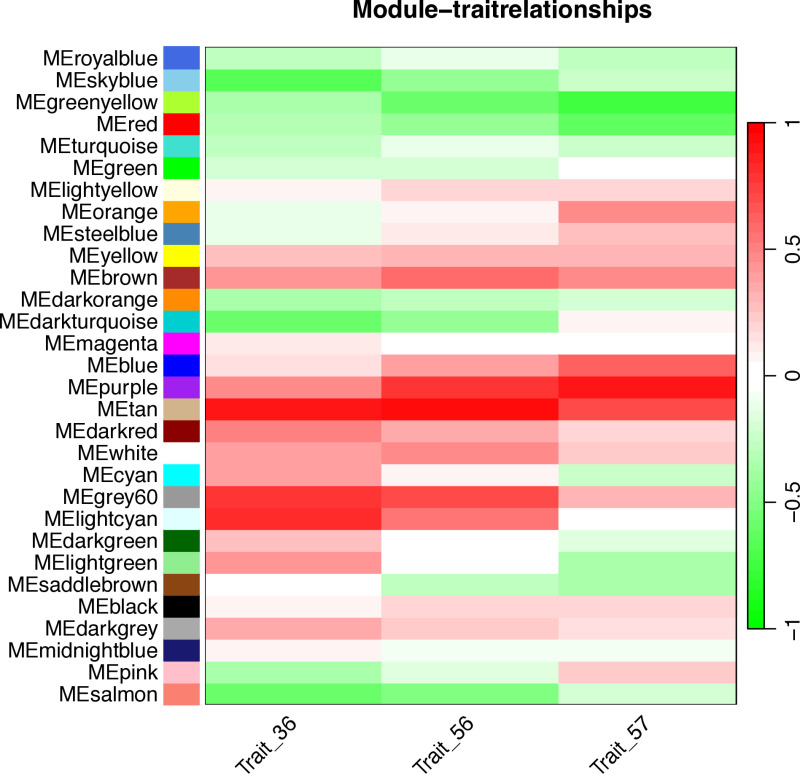
WGCNA module correlation heatmap with phenotypes. Each row corresponds to a module gene, and each column to a trait. The module name is displayed on the left side of each cell. The table is color-coded according to the correlation legend. The right side of the heatmap indicates the strength and direction of the correlations (red for positive correlation, green for negative correlation). Trait_56 represents the data of first field observation trial, Trait_36 represents the data of second field observation trial, and Trait_57 represents the data of in vitro pathogen inoculation trial.

**Fig 7:**
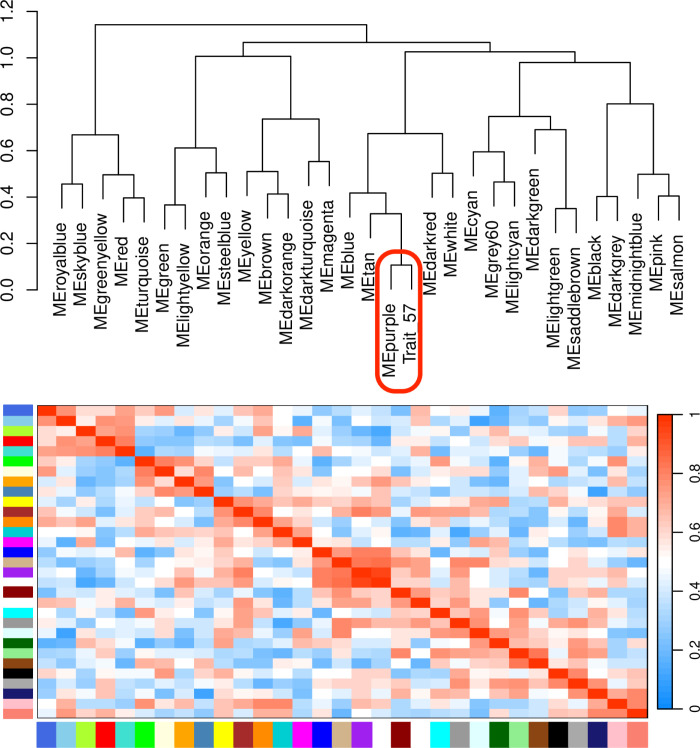
Hierarchical clustering dendrogram and heatmap of eigengenes. Red represents high adjacency (positive correlation), and blue represents low adjacency (negative correlation). Trait_57 represents the data of in vitro pathogen inoculation trial. Both the dendrogram and the heatmap show that the purple module is highly correlated with Trait_57.

Correlating the 30 merged modules with phenotypic data revealed that the purple module exhibited the strongest positive correlation with the target trait, while the yellow-green module showed the most significant negative correlation (Fig 6). In addition, by performing hierarchical clustering on the merged modules, it was observed that the 30 clusters were grouped into six major clusters, each containing two branches (Fig 7). Notably, the purple module co-clustered with Trait_57, suggesting that the expression patterns of genes within the module are convergent with phenotypic variation. Based on these results, we identified the purple and yellow-green modules as the key candidates associated with the target resistance traits.

### 3.5 Gene localization of candidate module genes

Using Bowtie program, we aligned the 4,217 SNP markers from the SNP genetic map to genes within the candidate modules. The results showed that a total of 23 SNPs were mapped to the genes in the purple module, and 16 SNPs were mapped to the genes in the yellow-green module (Table 5). Genes from the purple module were distributed across eleven linkage groups (LGs), while those from the yellow-green module spanned nine LGs. Only LG01 and LG02 lacked genes from both modules.

**Table 5:** The summary of gene localization of purple and yellow-green module genes.

Gene name^*^	Module	SNP_markers	Position (cM)	Linkage group
*GWHGACFB000274*	purple	*h4*	20.05	LG03
*GWHGACFB009129*	purple	*f968*	21.84	LG03
*GWHGACFB001233*	purple	*dm90*	85.55	LG03
*GWHGACFB001532*	purple	*f1683*	103.62	LG03
*GWHGACFB001749*	purple	*m1047*	107.93	LG03
*GWHGACFB001657*	purple	*f1895*	112.1	LG03
*GWHGACFB003983*	purple	*f355*	62.38	LG04
*GWHGACFB003017*	purple	*f462*	15.53	LG06
*GWHGACFB007317*	purple	*m1708*	76.37	LG07
*GWHGACFB000443*	purple	*f1277*	19.72	LG08
*GWHGACFB003565*	purple	*m286*	95.1	LG08
*GWHGACFB003531*	purple	*df573*	59.36	LG09
*GWHGACFB007895*	purple	*m329*	8.6	LG10
*GWHGACFB001992*	purple	*df750*	80.38	LG10
*GWHGACFB007475*	purple	*f1622*	62.66	LG11
*GWHGACFB002788*	purple	*h57*	13.88	LG12
*GWHGACFB003076*	purple	*f1289*	32	LG12
*GWHGACFB003662*	purple	*m1659*	59.71	LG12
*GWHGACFB003667*	purple	*df323*	63.41	LG12
*GWHGACFB006759*	purple	*m1901*	14.17	LG14
*GWHGACFB006235*	purple	*m574*	52	LG14
*GWHGACFB009932*	purple	*m1256*	11.42	LG15
*GWHGACFB010318*	purple	*m792*	41.69	LG15
*GWHGACFB000021*	yellow-green	*m104*	7.6	LG03
*GWHGACFB000823*	yellow-green	*f366*	56.18	LG03
*GWHGACFB000834*	yellow-green	*m576*	58.77	LG03
*GWHGACFB000964*	yellow-green	*m407*	55.01	LG05
*GWHGACFB003302*	yellow-green	*dm875*	83.3	LG07
*GWHGACFB006147*	yellow-green	*df443*	84.27	LG08
*GWHGACFB006749*	yellow-green	*h216*	101.49	LG08
*GWHGACFB008699*	yellow-green	*m659*	20.99	LG11
*GWHGACFB004704*	yellow-green	*m1553*	55.27	LG11
*GWHGACFB004314*	yellow-green	*f1498*	73.52	LG11
*GWHGACFB002946*	yellow-green	*f1090*	21.85	LG12
*GWHGACFB009712*	yellow-green	*dm412*	81.73	LG13
*GWHGACFB006555*	yellow-green	*h253*	27.38	LG14
*GWHGACFB006018*	yellow-green	*m236*	61.4	LG14
*GWHGACFB003821*	yellow-green	*df54*	49.39	LG15
*GWHGACFB008404*	yellow-green	*dm711*	69.63	LG15

* Gene names are derived from the ‘*Longjing 43*’ reference genome.

### 3.6 Identification of Key trait-associated Genes through WGCNA-QTL Co-Localization

**Fig 8:**
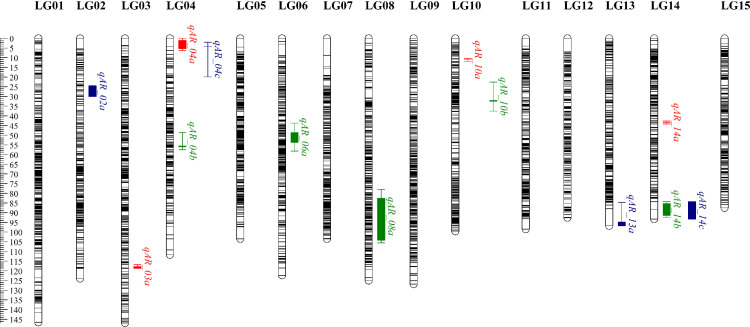
QTLs interval for thirteen anthracnose resistance-related traits. The green, red, and blue intervals represent the QTL regions identified for traits_36, traits_56, and traits_57, respectively.

As illustrated in Fig 8., we identified 13 QTLs associated with the target traits. These QTLs were spread across seven LGs (LG 02, LG 03, LG 04, LG 06, LG 08, LG 10, and LG 14), with LOD scores ranging from 3.07 to 10.52. The highest LOD score was observed for the QTL *qAR_14c* on LG 14 (10.52), while the lowest LOD score was for the QTL *qAR_04a* on LG 04 (3.07). Each of the 13 QTLs accounted for 4.50 to 43.50% of the phenotypic variation (as indicated by the contribution rate R^2^%). Except for the four QTLs associated with Trait_36 (*qAR_14a*, *qAR_03a*, *qAR_10a*, and *qAR_04a*), all other QTLs were considered major QTLs (with a contribution rate greater than 10%), and among them, three major QTLs had a contribution rate greater than 30%. The QTL *qAR_14c* on LG 14 had the highest phenotypic contribution rate, accounting for 43.50% of the phenotypic variation, whereas the QTL *qAR_10a* on LG 10 had the lowest phenotypic contribution rate, accounting for only 4.50% of the phenotypic variation.

**Fig 9:**
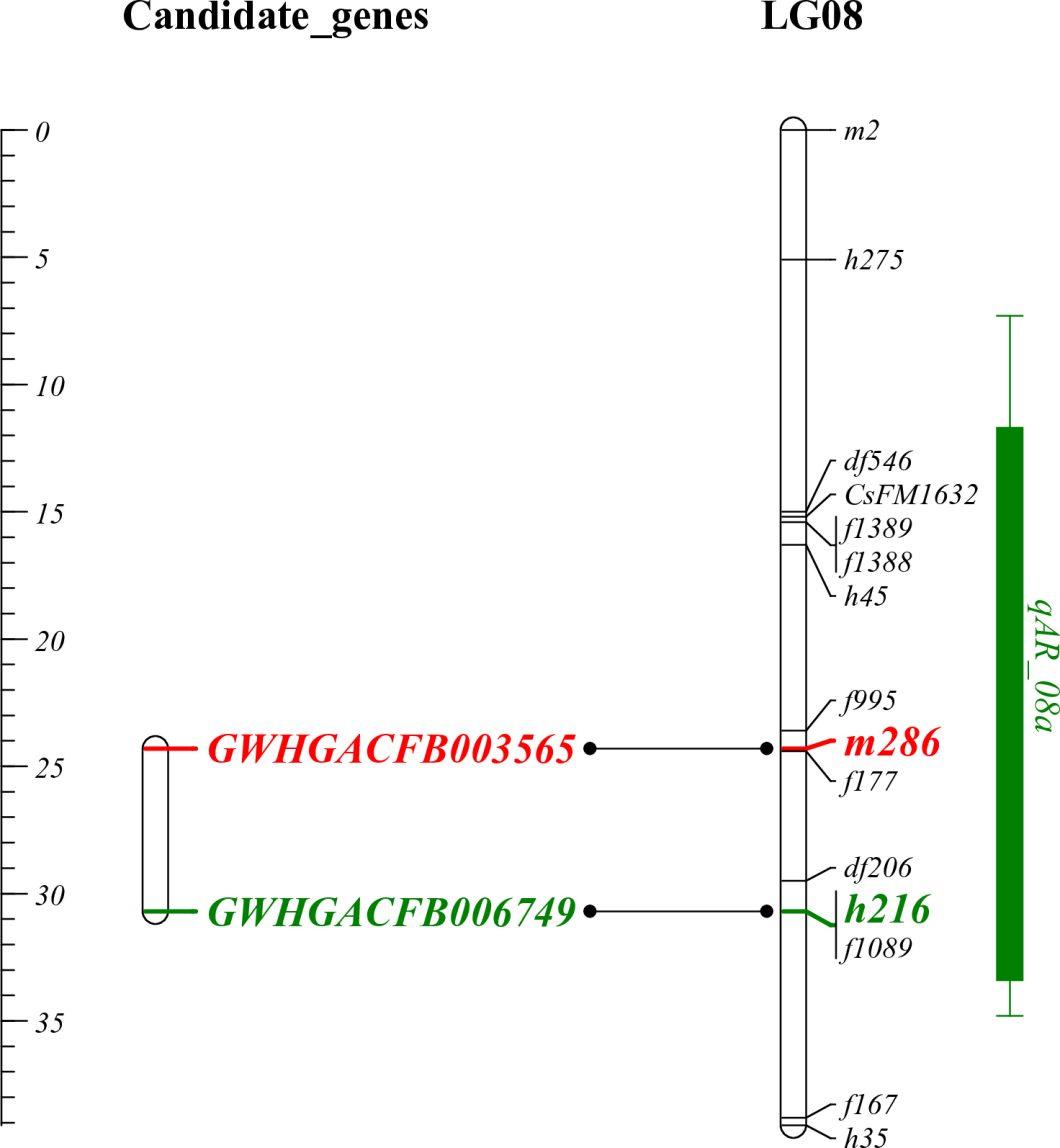
Homologous between genes within candidate modules and markers within QTL intervals of LG08.

By integrating the genomic positions of candidate module genes (Table 5.1 and 5.2) with the QTL intervals of the target traits (Table 6), we found that the gene (*GWHGACFB003565*) within the purple module and gene (*GWHGACFB006749*) within the yellow-green module can be mapped to positions 95.1 cM and 101.49 cM, respectively. Both loci were located within the LG08 QTL interval (Fig 9), thereby identifying these genes as key genetic determinants for the target traits.

**Table 6:** QTLs Interval for Targeted Phenotypic Traits.

Traits^*^	QTL name	LGs	Position	Marker	LOD	R %
Trait_36	*qAR_14a*	LG14	43.34	dm627	3.25	6.2
	*qAR_03a*	LG03	118.46	m1642	3.24	4.6
	*qAR_10a*	LG10	10.86	df375	3.21	4.5
	*qAR_04a*	LG04	4.29	MSE0226	3.07	5.7
Trait_56	*qAR_14b*	LG14	88.44	m969	6.28	37.6
	*qAR_08a*	LG08	102.84	m549	5.42	13.7
	*qAR_10b*	LG10	32.26	m818	4.49	12.2
	*qAR_06a*	LG06	48.67	df134	4.46	12
	*qAR_04b*	LG04	55.89	df498	4.37	11.3
Trait_57	*qAR_14c*	LG14	88.44	m969	10.52	43.5
	*qAR_04c*	LG04	4.29	MSE0226	4.12	30.5
	*qAR_13a*	LG13	96.74	f1003	4.06	13.8
	*qAR_02a*	LG02	24.52	df1016	3.77	10.5

* Trait_56 represents the data of first field observation trial, Trait_36 represents the data of second field observation trial, and Trait_57 represents the data of in vitro pathogen inoculation trial.

**Fig 10:**
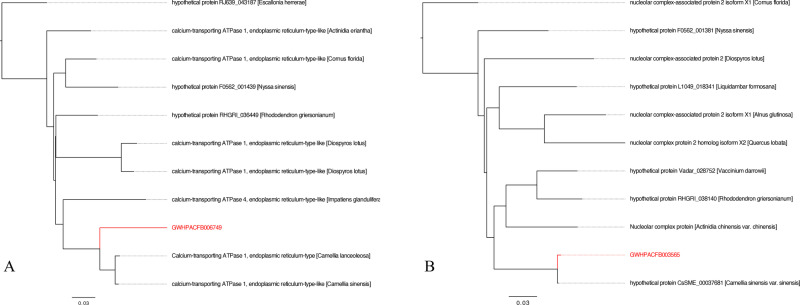
Phylogenetic Tree of Two Key Genes Based on NCBI BLAST Homology Analysis. (A) represents Gene (*GWHGACFB003565*), (B) represents Gene (*GWHGACFB006749*).

Functional annotation analysis was performed on the protein sequences of the reference genome to determine the potential functions of the candidate genes. As shown in S1 Table, Gene (*GWHGACFB003565*) was functionally annotated as Nucleolar complex protein 2 homolog (*NOC2L*); Gene (*GWHGACFB006749*) was functionally annotated as the calcium transport ATPase 1. Additionally, the sequences of the two genes were subjected to NCBI BLAST homology analysis, and the results (Fig 10) were consistent with the functional annotation findings.

### 3.7 Validation of Candidate Genes by qRT-PCR

**Fig 11:**
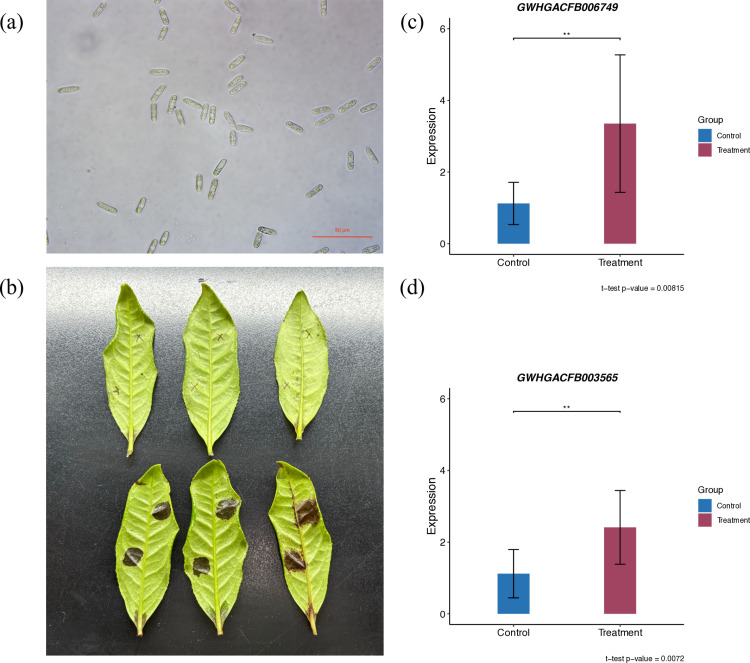
(a) The spore morphology of the pathogenic microorganism for inoculation. (b) lesion morphology in both control and treated groups at 7 days post-inoculation. (c) Relative expression levels of candidate gene *GWHGACFB006749*. (d) Relative expression levels of candidate gene *GWHGACFB00356*. (**p < 0.01).

To validate the reliability of transcriptome data, we selected fresh leaves of cultivar ‘FD’ as experimental materials. After wounding with sterile insect pins, leaves were divided into two groups: control (sterile water treatment) and treatment (spore inoculation). At 7 days post-inoculation, qRT-PCR analysis showed that while control wounds exhibited no visible water-soaked lesions, treated wounds developed characteristic anthracnose lesions (Fig 11b). Candidate genes *GWHGACFB003565* and *GWHGACFB006749* exhibited significant upregulation (Fig 11c–11d) in treated samples (*p* < 0.01), with *GWHGACFB003565* showing 2.41 ± 1.03-fold and *GWHGACFB006749* 3.35 ± 1.92-fold increases relative to controls (normalized to *CsGAPDH*). These results preliminarily demonstrate that the candidate genes identified in this study possess potential roles in regulating anthracnose resistance in tea plants.

## 4. Discussion

### 4.1. Integrated WGCNA-QTL co-localization strategy for candidate genes identification

In natural environments, plants have evolved sophisticated immune system to defend against pathogen infections [40]. Under biotic stress, resistant plants typically recognize pathogen virulence factors (effectors) through effector-triggered immunity (ETI), which leads to a robust resistance response. To defend against infections, plants employ nucleotide-binding site, leucine-rich repeat (NBS-LRR) proteins to intercept pathogen effectors and inhibit pathogen growth [41]. Consequently, plant resistance phenotypes generally arise from polygenic interactions.

In traditional molecular breeding strategies, there are two primary strategies for the genetic dissecting complex quantitative traits controlled by multiple genes: whole-genome scanning and the candidate gene approach [42], each with distinct advantages and limitations. In general, genome-wide scanning only locates the glancing chromosomal regions of quantitative trait loci (QTLs) at cM-level with the aid of DNA markers under family-based or population-based experimental designs, which usually embed a large number of candidate genes [43].

In tea plant research, QTL and WGCNA both have applications in the identification of functional genes [44,45], yet inherent constraints exist. Tea plants are self-incompatible [46], and their genetic populations often consist of F_1_ progeny, which results in larger QTL intervals that are not benefit to fine mapping [47,48], whereas WGCNA trait-associated modules often contain hundreds of co-expressed genes [19–21]. To address these limitations, in our study, we developed an integrative approach mapping WGCNA modules genes onto high-density SNP genetic maps and selecting those co-localizing with QTL intervals. In the end, we identified one candidate gene within each of the two trait-associated WGCNA modules. Our attempt also has provided a novel strategy for the identification of functional genes in the tea plant.

### 4.2. Functions implications of candidate genes in anthracnose resistance

The candidate gene (*GWHGACFB003565*) from the purple module was functionally annotated as *NOC2L*, a known inhibitor of histone acetylation. Specifically, *NOC2L* functions as an inhibitor of histone acetyltransferase (INHAT) by binding histone tails to block HAT-histone interactions [49]. Notably, histone acetylation dynamics are directly implicated in plant immunity [50–52]. For instance, in tomato, infection by *Ralstonia solanacearum* leads to an increase in the level of histone acetylation in resistant varieties [53]. Thus, we hypothesize that this candidate gene may mediate tea plant responses to *Colletotrichum* infection through histone acetylation suppression. Here, we demonstrate that *GWHGACFB00356* exhibits significant upregulation in *Colletotrichum*-inoculated samples compared to controls (Fig 11.d, *p* < 0.01, 2.41 ± 1.03-fold), indicating its responsiveness to anthracnose infection and potential functional role in host defense mechanisms.

The perception of pathogen invasion by plants triggers the response of pattern recognition receptors (PRRs), which activate the influx of extracellular calcium ions (Ca^2+^) into the cytoplasm (Ca^2+^ burst). Concurrently, Ca^2+^/ CaM regulates the synthesis of downstream signaling components, such as nitric oxide (NO) and hydrogen peroxide (H_2_O_2_), which are crucial for the development of the Hypersensitive Response (HR) [54]. This suggests that genes responsible for initiating and regulating downstream calcium (Ca^2+^) signaling events during plant defense responses to pathogens play a crucial role in their immunity. The candidate gene (*GWHGACFB006749*) from the yellow-green module encodes a calcium transport ATPase 1. Previous studies have demonstrated that during pathogen-triggered plant immune responses, calcium transport ATPases can modulate the transport of Ca^2+^ across the tonoplast membrane, thereby affecting the associated defense reactions [55,56]. Our results confirm significant upregulation of this gene following Colletotrichum infection (Fig 11c, *p* < 0.01, 3.35 ± 1.92-fold). We therefore propose that it modulates tea plant resistance by orchestrating Ca^2+^ homeostasis, potentially through regulating defense-related Ca^2+^ signatures and maintaining tonoplast Ca^2+^ fluxes during immune responses.

Currently research on tea plant anthracnose resistance implicates multiple pathways, including auxin signaling, ROS scavenging pathways, salicylic acid-mediated defense, receptor-like kinases, and the regulation by transcription factors [57]. Notably, Jeyaraj et al. discovered through transcriptome analysis of varieties with differential resistance to anthracnose that calmodulin-binding proteins (CBP) and Ca^2+^ -dependent protein kinases (CDPK) are differentially regulated by miRNAs [58]. While calcium transport ATPases and *NOC2L*-mediated epigenetic regulation remain understudied in tea-anthracnose interactions, the candidate genes (*GWHGACFB006749* and *GWHGACFB003565*) identified in our work establish novel mechanistic avenues for future investigation.

## 5. Conclusions

In this study, we evaluated anthracnose resistance across eight samples, observing significant variation in susceptibility to *Colletotrichum* infection. Subsequently, through WGCNA analysis of transcriptome data, we identified 30 co-expression modules, two of which showed significant correlations with resistance phenotypes. We performed WGCNA-QTL co-localization analysis mapped two candidate genes (*GWHGACFB003565* and *GWHGACFB006749*) within LG08 QTL intervals. After functional annotation, these two genes encode a histone acetylation regulator (*NOC2L*) and a calcium transport ATPase 1. Both genes have established roles in plant immunity. qRT-PCR validation demonstrated their significant upregulation in *Colletotrichum*-infected tea leaves, confirming their infection-responsive expression patterns. Additionally, we further enhanced the functional annotation of the ‘*Longjing 43*’ reference genome. These findings provide valuable insights for anthracnose-resistant tea breeding. Future work requires development of a reliable genetic transformation system for *C. sinensis* to characterize candidate gene functions during infection and establish molecular markers for breeding applications.

## Supporting information

S1 Table Functional annotation of the reference genome protein sequences by EggNOG 6.0. (XLSX)
